# Supplementary material for: Improved prediction of hiking speeds using a data driven approach
Source: PLoS One. 2023 Dec 18;18(12):e0295848. doi: 10.1371/journal.pone.0295848 (PMC10727444; doi:10.1371/journal.pone.0295848)
Supplement: S1 File — (PDF) [file pone.0295848.s001.pdf]

S1 Supporting Information. Data sources

Data sources table

Table 1. Summary of data sources used during this work

Data Type	Data Source	Download Date	Notes
Hikr GPS data	Hikr.org	01-07-2021	Within the UK data[1], only tracks which took place within Scotland[2] were used for exploratory study (see S3 Supporting Information).
OpenStreetMap GPS data	OpenStreetMap.org	01-07-2021	Accessed using planet.gpx regional extracts[3]
Ordnance Survey elevation data	Ordnace Survey Terrain 5 DTM	05-08-2021	Accessed using EDINA Digimap Ordnance Survey Service[4]
OpenStreetMap road data	OpenStreetMap.org	04-08-2021	Accessed using planet.osm regional extracts[5]
England lidar data	National LIDAR Programme	16-09-2021	2m resolution data was used and accessed using EDINA LIDAR Digimap Service[4]
Wales lidar data	LIDAR terrain and surfaces models Wales	16-09-2021	2m resolution data was used and accessed using EDINA LIDAR Digimap Service[4]

Note: In regions where lidar data was available as part of both the England and Wales lidar datasets, the data values from England were used.
[1] `https://www.hikr.org/region516/ped/?gps=1`
[2] `https://www.hikr.org/region518/ped/?gps=1`
[3] `http://zverik.openstreetmap.ru/gps/files/extracts/europe/great_britain.tar.xz`
[4] `https://digimap.edina.ac.uk`
[5] `http://download.geofabrik.de/europe/great-britain.html`

Code Availability

Documented code written for this study is available online in a Github repository `AndrewWood94/PhDThesis`, and is licensed under the terms of the GNU General Public License v3.0.
