# Supplementary material for: Improved prediction of hiking speeds using a data driven approach
Source: PLoS One. 2023 Dec 18;18(12):e0295848. doi: 10.1371/journal.pone.0295848 (PMC10727444; doi:10.1371/journal.pone.0295848)
Supplement: S2 File — (PDF) [file pone.0295848.s002.pdf]

# S2 Supporting Information. Data acquisition and preparation

## Importing GPS tracks

Approximately 20,000 tracks with GPS data were obtained, the majority of the tracks used here were from OpenStreetMap (OSM) [1], with a second, smaller set from Hikr.org [2]. The OSM data consisted of a GPS data dump of all tracks on OpenStreetMap which contain points within the UK, as of April 2013 (the date of the last GPS data dump by OSM). This dataset does not contain any information about the mode of transport being used when the track was recorded, or the device used for the recording. The Hikr dataset contained all of the Hikr reports which were uploaded from July 2009 to July 2021. Although only relatively small quantities of Hikr data are available, all of the tracks used were explicitly tagged as hiking reports, so could be used as the basis of a filter to determine which of the OSM tracks contained walking data (see Data Filtering).

Each GPS track is made up of track segments, where each track segment is a continuous run of points; a point is a data pair containing the device location and time of recording. The exact nature of tracks and track segments depends on the individual device settings being used. Some devices record points at fixed time intervals, others may only record a new point after travelling a sufficiently large distance away from the previous point. In general, a new track or a new track segment is created whenever the device is switched off or loses signal for a period, although this is not always the case.

All track segments which were fully contained within the area covered by the OS Terrain 5 DTM were imported (see Elevation and Slope). The GPX files were read using a customised version of the `GPX Segment Importer` plugin for QGIS [3], extended to also read elevation and terrain information (code on Github - `AndrewWood94/PhDThesis`). Although a single GPX file can contain multiple separate tracks, we considered all data within a file to be part of the same track. Tracks within the same file were highly likely to be undertaken by the same individual, and thus have correlated walking speeds. To account for the variance caused by different hike locations and difficulty, each track segment within a file was processed individually.

For each track segment, the list of points was converted to a series of connected linestrings, with the following properties calculated and attached to each one:

- Start coordinate
- End coordinate
- Start time
- Duration
- Distance
- Speed

We took a decision to exclude very short tracks (less than 250m in length of 2.5 minutes in duration as well as any tracks where the median speed was greater than 10km/h, as it was felt they might not be a reliable representation of a real walk.

Duplicate track segments (those which contained a section with the same start and end coordinates, the same starting date and time, and the same duration) were tagged, and only the first instance of each was retained. It is believed that these duplicates were a result of either users uploading the same track multiple times, or GPS devices recording multiple versions of the same track with different levels of accuracy or

automatic filtering. Finally, a small number of outlier Hikr tracks were checked manually and removed as looking at the metadata or track description revealed that the routes in question were labelled as trailruns, and so should not be considered.

### Elevation and Slope

The Ordnance Survey Terrain 5 Digital Terrain Map (DTM) [4] provides the elevation over the whole of Great Britain at 5 m intervals, with an accuracy of greater than 2.5 m RMSE. After reading in each track, the DTM was used to calculate the following:

- Elevation
- Walking slope
- Hill slope

Although the GPX files also contained elevation data which could be used to calculate the walking slope, there were a number of reasons to prefer the Ordnance Survey data:

- Multiple tracks were found where the elevation was measured to the nearest half metre or metre on the GPS device. Using this value would lead to rounding errors in slope calculations over short distances. The Ordnance Survey values for elevation have a higher level of precision which could alleviate these errors.
- Using OS values avoids any concern about potential discrepancies arising from differences in calibration across GPS devices, so we could ensure that the same location on different tracks would have the same elevation.
- We were also investigating the impact of hill slope, which could not be calculated from the GPS track data, and using the same data source for both calculations should allow for better evaluation of interactions between the two variables.

When first reading each data track, the elevation DTM was sampled to provide the spot height of the start co-ordinate, and the hill slope at this point was calculated using the quadratic surface method [5, 6]. As explored in previous work [7], this method produces the most accurate slope estimates, especially given the high resolution of the DTM being used for calculations. The walking slope was calculated using the spot heights of the start and end coordinates of each linestring, and the distance between those points.

### Roads and Paths

Road and path data from OpenStreetMap was downloaded from GeoFabrik.de [8] and contains the data as of August 2021. Although similar data is available from Ordnance Survey, the OSM dataset was preferred due to it providing a more detailed classification than Ordnance Survey, in terms of the road or path type.

The OSM road and path data is made up of linestrings (single vector lines, as opposed to area features which match the width of the road) and it would be very unlikely for all of the points on a GPS route which follows a road to fall exactly along that line, making it difficult to determine which points should be classified as following the road or path. This problem was then exacerbated by two factors; inaccuracies within the GPS readings, and inaccuracies within the map data itself. This combination of potential errors meant that we needed to decide upon a radius around each point to search for roads or paths.

Here we classified a point as being on-road if a single feature of the OSM road dataset was found within a 50 m radius around the point. This radius was determined after a number of iterations.

This inclusive distance will lead to an over classification of on-road points. Due to the relative numbers of on-road vs off-road points, we decided it would be preferential to mis-classify points as being on a road rather than the other way around, as this would have a much smaller impact on any resulting models.

By consulting the descriptions of the OSM road-type definitions [9], we could separate the on-road points into two categories, paved and unpaved. When doing this, we assumed that the standard road type was paved. Therefore, if a point contained multiple road types, we only considered it to be unpaved if none of the road types detected were paved. The paved road types are the following:

- Cycleway
- Footway
- Living_street
- Motorway
- Motorway_link
- Pedestrian
- Primary
- Primary_link
- Residential
- Secondary
- Secondary_link
- Service
- Steps
- Tertiary
- Tertiary_link
- Trunk
- Trunk_link
- Unclassified
- Unknown

While these are unpaved:

- Bridleway
- Path
- Track
- Track_grade1
- Track_grade2
- Track_grade3
- Track_grade4
- Track_grade5

**Obstruction Height**

For England and Wales, a lidar Digital Terrain Map (DTM) and Digital Surface Map (DSM) were downloaded (see S1 Supporting Information), both at 2 m resolution, with an accuracy of 15 cm RMSE [10–12]. The DTM gives the ground height above sea level every 2 m, while the DSM provides the surface height (i.e. taking into account buildings or trees etc). For Scotland, there is limited coverage in more rural (i.e. off-road) areas, and most of the available data is at very high resolution (25-50 cm). This is generally greater than the accuracy of the GPS devices used. Given the partial coverage and resolution mis-match in the Scottish datasets, further analysis was limited to tracks in England and Wales.

The start coordinate for each point in a GPS track was sampled in both the DTM and DSM, and the difference between the two values taken as the level of terrain obstruction for the point. For example, a point in a woodland may have a DTM height of 80 m above sea level, and a DSM height of 85 m (the height at the top of the tree canopy), giving us a terrain obstruction value of 5 m.

## Break Finding

For this work, we were interested in calculating the active components of a route (as opposed to breaks or rests), as that is the data on which a walking speed model should

be based. Performing analysis on the data without first removing breaks would likely result in inaccurate and lower estimates for the movement speed. Similarly, when developing the filter to find walking routes in the OpenStreetMap dataset we wanted to ensure that we were only considering the active route components.

Initial inspection of the walking tracks showed that a large number contained obvious breaks, while the device was still recording. The simplest method to find breaks, and the first implemented, was to tag all points where there was no movement (i.e. walking speed = 0 km/h). The next step was to tag any individual points which represented over 1 km or 3 minutes of travel as breaks. These points could occur in areas where the device lost signal for a period. Although this should result in the creation of a new track segment, there were a number of tracks where this was clearly not the case. Points with speeds >10 km/h occurring immediately following a long (>3 minute) point were also tagged as breaks. Situations like this occurred on a number of devices, likely when a device automatically paused recording for a break. Once significant movement was detected, two points were then added in quick succession, the first at the original location with the time the movement started again, and the second at the new position of the device. This resulted in one high duration point with very little movement, followed by single-second duration movement with high speed, where the device 'caught up' with the correct location.

Unfortunately, this did not capture all of the break points due to GPS drift; the distance between the measured position of the GPS device and the true location. This error is always present, but is more obvious when stationary, as a large number of points are recorded in the same general area, forming clusters. Examples of these clusters can be seen in Fig 2 of the main paper.

Although these locations are easy to identify when the route is visualised, we needed an automatic filter in order to remove them from our analysis. The movement speeds calculated from these drift points can vary greatly, depending on the sampling rate of the device and the amount of GPS drift. A drift measurement of 6 m in a very short time (1-2 seconds) would mean a very high speed (10-20 km/h) is found, but a series of very low speeds in a row could be due to a break with a small amount of drift, or it could indicate a particularly difficult (and therefore slow) section of the route.

A number of previous studies explored methods to classify GPS tracks into activity types [13–20], however none could be directly applied to this problem. This is because they were usually trying to identify different modes of transport, which can be clearly distinguished by different travel speeds. However, as mentioned above, the speed measurements caused by GPS drift can easily be in the same range as an expected walking speed. Methods which look for clusters similar to those being investigated here were also not useable as they generally require a known sampling rate for the GPS device [18], or involve checking clusters against a pre-existing database of features [19, 20]. Our data consisted of tracks created using a wide range of devices and settings, and over a very large area, so it was not possible to either assume a fixed sampling rate, or to pre-select features where breaks were likely. Instead, various ideas from a number of works were combined and adapted to find clusters of points, which were then checked to see if they should be identified as a break.

Firstly, based on [20], a modified version of DBSCAN [21] was used to identify clusters. Unlike in the standard algorithm, each point only looked for neighbours whose timestamp was within 10 minutes of the point being checked. This prevented clusters being found on routes which doubled back on themselves or contained loops. Secondly, as sampling rates were not consistent across devices and the tracks covered a variety of terrains, we could not assume a fixed radius to find neighbouring points. Instead the median travel distance for the particular track segment being investigated was used ($r_{median}$).

*DEFINITION 1. Neighbourhood of a point: Let $\{p_0, p_1, ..., p_n\}$ be points on a GPS track segment, with timestamps $\{t_0, t_1, ..., t_n\}$, a median distance $r_{median}$ between consecutive points and a median point speed $s_{median}$. The neighbourhood $N_k$ of a point, $p_k$, is the set of points $p_i$ such that:*

$$dist(p_i, p_k) < r_{median} \text{ and } |t_i - t_k| < 600s$$

Using these conditions, all points along each track segment were tested to find point clusters.

*DEFINITION 2. Point cluster: A point cluster, C, is formed from a point neighbourhood, $N_k$, if any of the following hold:*

1. *At least 5 non-consecutive points are found in $N_k$*
2. *At least 10 consecutive points are found in $N_k$*
3. *A point within $N_k$ has a 'high speed'; a speed greater than $2 * s_{median}$*
4. *A point $p_k$ is immediately preceded by a 'high speed' point*
5. *A point within $N_k$ has a 'very low speed'; a speed less than 0.01 km/h*

Examples of each of these conditions are shown in Fig 1A. The first two conditions worked together to prevent finding clusters in unusually slow sections of a route, such as a steep hill climb, but allowed for areas where GPS drift was small and an entire cluster was contained within $N_k$. The third and fourth conditions found 'high speed' points which occurred when a large amount of GPS drift was measured, and could spread a cluster out over a wider area. Without accounting for these points separately, the algorithm would often end up registering a single break as multiple short breaks separated by high speed movements. The fifth condition was necessary for situations where the device was set to only record new points once a minimum distance has been travelled from the previous location. Note that in the third and fifth cases in definition two, the point immediately following the high- or low-speed point was added to the cluster, even if it was not included in $N_k$. Similarly, in the fourth case, the cluster included the preceding high-speed point regardless of whether it was in $N_k$.

Once a point cluster was identified, each point within it was tested and any further clusters found were added to the original. This continued recursively until every point within the cluster had been checked and no new clusters were found. This allowed us to identify clusters (and breaks) which lasted longer than the 10 minute Neighbourhood threshold, as each point added to a cluster allows the search window to be extended beyond the initial limit.

Following this, further steps were taken to try and ensure that only legitimate breaks were classified, rather than slow sections of movement. Firstly, each point within the cluster was tested for 'break likelihood' using a simple classification methodology based on the approach used by Wan and Lin [17], which uses two variables; the point speed, $s_i$, and the point angle, $\alpha_i$.

*DEFINITION 3. Point speed: The speed, $s_i$, at a point, $p_i$ is categorised as follows:*

- *Low: $s_i < s_{median}/2$*
- *Medium: $s_{median}/2 < s_i < 10m/s$*
- *High: $s_i > 10m/s$*

*DEFINITION 4. Point angle: The point angle, $\alpha_i$, for a point, $p_i$, is the angle created at $p_i$ by the lines connecting it to $p_{i-1}$ and $p_{i+1}$. It is then categorised as follows:*

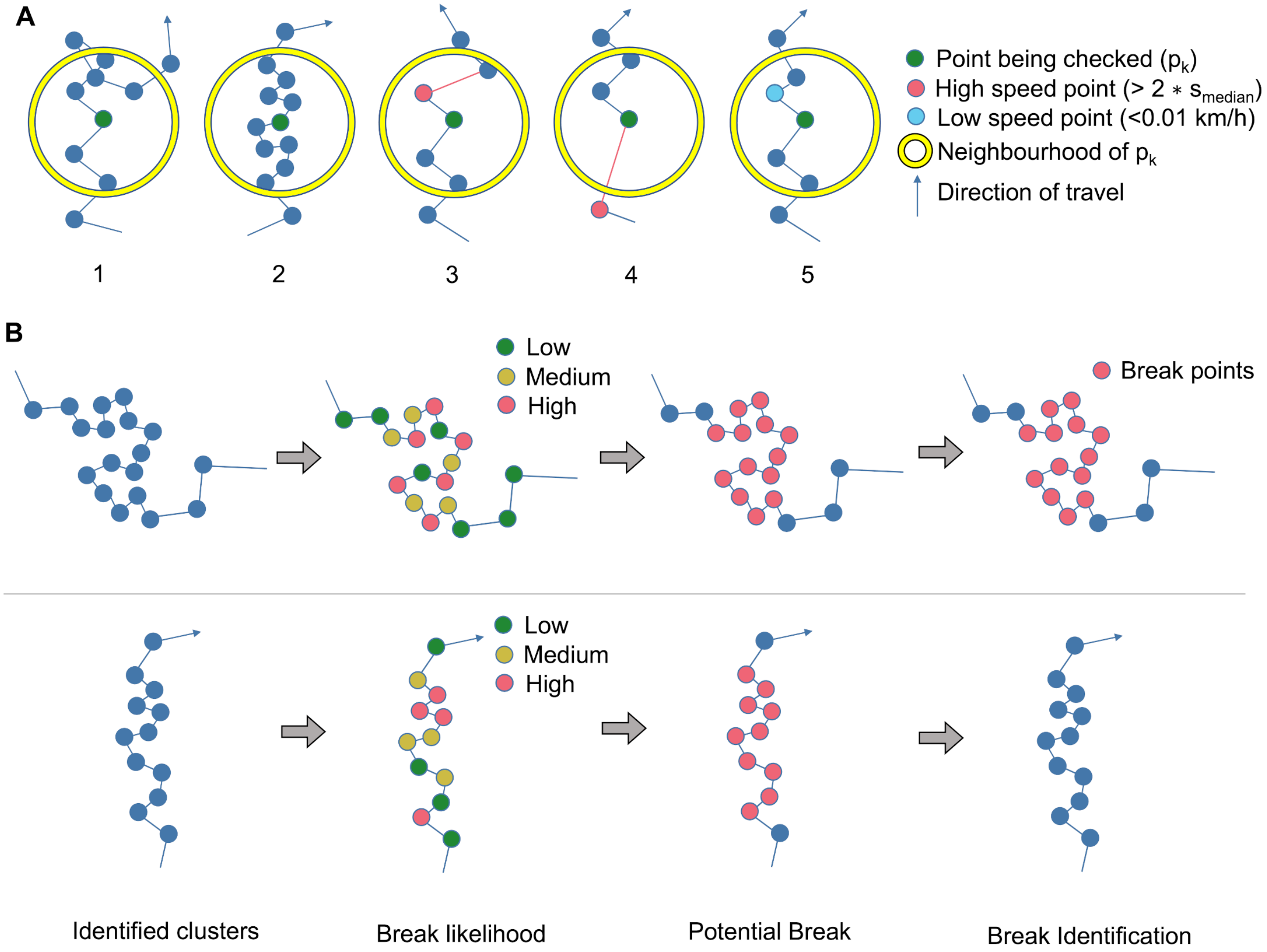


**Fig 1. Demonstration of how breaks are identified from point clusters.** (A) The five scenarios in which a point cluster is identified around a point. (B) Points in a cluster are checked to find their break likelihood, and see if a potential break can be identified. Potential breaks are then checked to see if a break can be formed.

– *Narrow:* $\alpha_i < 90°$
– *Wide:* $\alpha_i >= 90°$

As discussed in [17], normal walking is unlikely to result in point angles below 90 degrees, unlike a stationary object subject to GPS drift which often produces narrower point angles. The break likelihood for each point in a cluster was found using the classifications in Table 1.

**Table 1.** Break likelihood classifications based on point speeds and angles

| | | Point Speed | | |
|---|---|---|---|---|
| | | Low | Medium | High |
| **Point Angle** | Narrow | High | Medium | High |
| | Wide | Medium | Low | Medium |

Once the break likelihoods were identified, the cluster was checked to find a potential break. Performing this step helped to limit the sizes of breaks, as walking points immediately preceding or following a break were often caught in the cluster.

*DEFINITION 5. Potential Break: A potential break, $B^*$, is created by ordering the points in a cluster and identifying the first and last points with a break likelihood of medium or high. All points in the cluster between these points (including the points themselves) form the potential break.*

As a break by definition implies no movement, any GPS drift in a given direction should be cancelled out by subsequent GPS drift in the opposite direction. For this reason, the bearing of all points within the potential break was found and assigned a quadrant, and a break was only formed if there was apparent motion in opposite quadrants.

$$\begin{aligned} Q_i = True\ iff \\ &\exists\ p \in B^* \mid (90 * (i-1))^\circ < p_{bearing} < (90 * i)^\circ, \\ &i = (1, 2, 3, 4) \end{aligned}$$

*DEFINITION 6. Break: A break, $B$, is created from a potential break, $B^*$, if both of the following hold:*

- $Q_1 = True$ *and* $Q_3 = True$, *or* $Q_3 = True$ *and* $Q_4 = True$.
- *Less than half the points in* $B^*$ *have a low break likelihood.*

Fig 1B provides examples of two point clusters and the process to identify breaks. Note that not every point in each cluster becomes part of the potential break; low break likelihood points at the ends of a cluster are excluded. After processing the second cluster, a break is not defined because the cluster does meet the requirement of apparent travel in opposite quadrants. While there is a substantial amount of east-to-west deviation in the track, it consistently heads north. Regions such as this identified in GPS tracks are more likely to indicate a period of challenging (and therefore slow) movement.

A substantial ground truth dataset of routes where the breaks are tagged does not exist, so we were unable to measure the accuracy of this break-finding algorithm numerically. However, a qualitative inspection of a small number of tracks suggested that it classified breaks well, with over-classification in some circumstances. This was preferable to under-classification for two reasons; firstly, as discussed below, only the longer breaks were actually removed from calculations so small breaks found as a result of over-classification have no impact. Secondly, we were performing analysis on very large datasets, so we expect the analysis to be robust to the incorrect removal of a small number of data points.

## Data Filtering

Before processing our data, we wanted to use information about walking speeds found in the Hikr data to identify and remove non-walking tracks from the OSM dataset. When doing this, we only wanted to compare the active component of each of the tracks. However, not all break points should necessarily be considered 'inactive'. The majority of the breaks seen in the dataset were under 30 seconds in length, and these were defined as 'micro-breaks'. Micro-breaks were felt to be a constituent part of the walk which most people would have to do, such as pausing to catch your breath, and were not excluded from further analysis. The exception to this was if the micro-break contained a single point with over 1 km of movement, or points with speeds of over 10 km/h. Speeds over 10 km/h in a region which had been identified as a break were very likely to be errors caused by GPS drift, and as such these points would not be useful in representing a typical walking speed. It was felt that 30 seconds is a reasonable length

such that breaks of this length or longer would be a conscious choice by the walker to stop, rather than being necessary for the route. After excluding long breaks, it was also decided to remove any breaks which occurred at the start or end of a track segment.

The remaining data were merged together into continuous sections at least 50 metres in length, to remove some of the variability caused by the GPS devices and elevation data resolution. Devices generally measure the time to the nearest second, so rounding errors over short distances could have a large impact on the estimated speeds, especially in combination with small inaccuracies in the location data. By merging the data together into longer sections the impact of these errors was greatly reduced, as any error made up a smaller percentage of the merged section. A similar inaccuracy existed with walking slope values. As our elevation DTM had a resolution of 5 m, a distance of under 5 m between two consecutive points could result in a walking slope of 0 degrees, regardless of how steep the terrain is in reality. By merging the points together, we smoothed out the steps in the data and produced a single datapoint with a slope value closer to the true value, which will help produce a model with more accurate walking speed predictions.

When merging data, the distance was taken as the cumulative distance across all points, and not the direct start point to end point distance. The merged point was classified as on a paved road if at least one of the constituent points was classified as being on a paved road. (Similarly a point was only classified as being off-road if none of the constituent points were on-road). The hill slope, walking slope and average obstruction height were calculated as the weighted average of the value at each datapoint, weighted by the duration of each point.

Once the data were merged into 50 m sections, any section with a speed above 10 km/h which was at the start or end of a segment, or next to a break point was considered to be part of the break. This was repeated recursively until no more high speed points were found next to breaks.

Although all of the Hikr data was tagged as a walk or hike, there were a small number of individual track segments with high average speeds. Upon further inspection it was clear that these were segments, within a larger walking track, where other modes of transport were used and should be filtered out, therefore segments with an average speed greater than 10km/h were removed.

For each remaining track segment the median, upper quartile and maximum speed were calculated, and statistics from these were found to use in filtering the OpenStreetMap data to remove non-walking tracks:

The upper quartile of the maximum Hikr speeds (approx. 6.0 km/h) (1)

The median of the median Hikr speeds (approx. 3.2 km/h) (2)

The upper whisker of the maximum Hikr speeds (approx. 8.1 km/h) (3)

The minimum of the upper quartile Hikr speed (approx. 2.3 km/h) (4)

The OpenStreetMap data were also merged into intervals of at least 50 m, while ignoring short micro-breaks. Similarly to the Hikr tracks, this dataset contained a number of tracks where there were clearly multiple movement methods, often when the user was driving or cycling to a hike location. Unlike in the Hikr data however, a change in transport method was often not accompanied by the start of a new track segment.

Instead, individual route segments appeared to contain a variety of transport methods, often separated by a number of very extreme points (likely where the device lost signal for a period). As we did not want to remove these segments entirely if they contained valid walking data, we used the extreme points as markers to break the segment down into smaller sections. The 'key points' were defined as the start and end points of the segment, as well as any point with a distance greater than 500 m, a duration greater than 3 minutes, or an apparent speed over 100 km/h (this is typically caused by a device refresh). After identifying the key points, the following conditions were applied:

- If only a single data point existed between a pair of key points it was ignored
- If the median speed between a pair of key points was greater than (1), then all points in the range were ignored

Following this, all segments were checked and the steps outlined below were carried out to remove unwanted data. These were repeated until no further data was removed.

1. If the segment contained less than 2.5 minutes or 250 m of useable data it was removed
2. Segments were removed if any of the following were true:
   - The median speed was greater than (1)
   - The minimum speed was greater than (2)
   - The upper quartile speed was greater than (3)
   - The upper whisker speed was less than (4)
3. All points with a speed above 10 km/h which were at the start or end of a segment, or next to a break point were considered part of the break.

For the remaining segments, the Hikr and OpenStreetMap data were then combined together into a single dataset. Visual inspection of the dataset revealed that the filters were good, but a small number of outliers remained. Therefore, the fastest and slowest 0.5% of the merged datapoints were removed (classified as breaks for further processing) and were not included in any modelling.

Following this a decision was made to remove data from tracks found in Scotland. Lidar data covering the walking tracks was necessary to model the terrain obstruction, and was not sufficiently available in Scotland at the time of the study. Furthermore, analysis showed that that walking speeds in Scotland were at the extreme end of what is seen throughout the rest of the UK (see S4 Supporting Information). Including this data without also including a corresponding extreme dataset where lidar data is available may result in incorrect modelling. All OSM track segments which took place within Scotland were excluded from further processing. Similarly Hikr tracks which were tagged as taking place in Scotland, and which fully took place in Scotland were excluded. Note that we defined tracks in Scotland as any track segments which were fully contained within the following OS grid squares:

HP HT HU HW HX HY HZ NA NB NC ND NF NG NH NJ NK NL NM NN NO NR NS NT NU NW NX NY

Within the NY tile, the following tiles were excluded:

09 19 29 39 49 59 69 08 18 28 38 48 58 07 17 27 37 47 06 16 26 36

A small number of Hikr tracks which took place in the Scottish islands were not removed as these were tagged separately on Hikr.org.

This left us with a final dataset of almost 88,000 km and over 7,600 tracks across the UK, with a mean speed of 4.64 km/h. All of the data remaining was assumed to consist

solely of hiking or walking tracks, although there are likely to be a number of areas where this was not the case. There is not a large difference in speed profile when walking or cycling up a steep incline, so further data filtering would require in-depth analysis of each individual track. There was no objective way to remove invalid points without potentially removing valid data as well. However, the volume of data used for modelling should alleviate errors arising from any non-hiking track segments remaining.

## References


1. OpenStreetMap contributors. OpenStreetMap.org; 2021. Using: Planet OSM regional extracts `http://zverik.openstreetmap.ru/gps/files/extracts/europe/great_britain.tar.xz`.

2. Hikr org. United Kingdom Hiking Reports; 2021. `https://www.hikr.org/region516/ped/?gps=1`.

3. Simon Gröchenig. QGIS GPX Segment Importer; 2019. Available from: `https://github.com/SGroe/gpx-segment-importer`.

4. Ordnance Survey (GB). OS Terrain 5 [ASC geospatial data], Scale 1:10000, Tiles: GB; 2020. Using: EDINA Digimap Ordnance Survey Service, (`https://digimap.edina.ac.uk`). Available from: `https://www.ordnancesurvey.co.uk/business-government/products/terrain-5`.

5. Zevenbergen LW, Thorne CR. Quantitative analysis of land surface topography. Earth Surface Processes and Landforms. 1987;12(1):47–56. doi:10.1002/esp.3290120107.

6. Dunn M, Hickey R. The effect of slope algorithms on slope estimates within a GIS. Cartography. 1998;27(1):9–15. doi:10.1080/00690805.1998.9714086.

7. Jones KH. A comparison of algorithms used to compute hill slope as a property of the DEM. Computers & Geosciences. 1998;24(4):315–323. doi:10.1016/S0098-3004(98)00032-6.

8. OpenStreetMap contributors. OpenStreetMap.org; 2021. Using: Planet OSM regional extracts: `http://download.geofabrik.de/europe/great-britain.html` note = Downloaded: 04-08-2021.

9. OpenStreetMap Wiki. Key:highway — OpenStreetMap Wiki,; 2022. Available from: `https://wiki.openstreetmap.org/w/index.php?title=Key:highway&oldid=2426496`.

10. Environment Agency. LIDAR Composite DSM 2017 - 2m [ASC geospatial data], Scale 1:8000, Tiles: England; 2017. Using: EDINA LIDAR Digimap Service, (`https://digimap.edina.ac.uk`). Available from: `https://www.data.gov.uk/dataset/fba12e80-519f-4be2-806f-41be9e26ab96/lidar-composite-dsm-2017-2m`.

11. Environment Agency. LIDAR Composite DTM 2020 - 2m [ASC geospatial data], Scale 1:8000, Tiles: England; 2020. Using: EDINA LIDAR Digimap Service, (`https://digimap.edina.ac.uk`). Available from: `https://www.data.gov.uk/dataset/a58f4e0d-27ba-440a-9a9c-274bc76500f5/lidar-composite-dtm-2020-2m`.

12. Natural Resources Wales. LiDAR Composite Dataset [ASC geospatial data], Scale 1:8000, Tiles: Wales; 2016. Using: EDINA LIDAR Digimap Service, (https://digimap.edina.ac.uk). Available from: http://lle.gov.wales/Catalogue/Item/LidarCompositeDataset?lang=en.

13. Zhou C, Jia H, Juan Z, Fu X, Xiao G. A Data-Driven Method for Trip Ends Identification Using Large-Scale Smartphone-Based GPS Tracking Data. IEEE Transactions on Intelligent Transportation Systems. 2017;18(8):2096–2110. doi:10.1109/TITS.2016.2630733.

14. Schuessler N, Axhausen KW. Processing Raw Data from Global Positioning Systems Without Additional Information. Transportation Research Record: Journal of the Transportation Research. 2009;2105:28–36. doi:10.3141/2105-04.

15. Biljecki F. Automatic segmentation and classification of movement trajectories for transportation modes; 2010. Available from: http://repository.tudelft.nl/view/ir/uuid%3A654587d2-6e93-4619-ab9a-29d95f843f35/.

16. Tsui S, Shalaby A. Enhanced System for Link and Mode Identification for Personal Travel Surveys Based on Global Positioning Systems. Transportation Research Record: Journal of the Transportation Research Board. 2006;1972:38–45. doi:10.3141/1972-07.

17. Wan N, Lin G. Classifying Human Activity Patterns from Smartphone Collected GPS data: A Fuzzy Classification and Aggregation Approach. Transactions in GIS. 2016;20(6):869–886. doi:10.1111/tgis.12181.

18. De Vries SI, Sterkenburg RP. Filtering GPS tracks: cluster detection, cluster classification and transportation mode classification; 2012. Available from: https://www.researchgate.net/publication/328131279.

19. Alvares LO, Bogorny V, Kuijpers B, de Macedo JAF, Moelans B, Vaisman A. A model for enriching trajectories with semantic geographical information. In: Proceedings of the 15th annual ACM international symposium on Advances in geographic information systems - GIS '07. New York, New York, USA: ACM Press; 2007. p. 1. Available from: http://portal.acm.org/citation.cfm?doid=1341012.1341041.

20. Palma AT, Bogorny V, Kuijpers B, Alvares LO. A clustering-based approach for discovering interesting places in trajectories. In: Proceedings of the 2008 ACM symposium on Applied computing - SAC '08. New York, New York, USA: ACM Press; 2008. p. 863. Available from: http://portal.acm.org/citation.cfm?doid=1363686.1363886.

21. Ester M, Kriegel HP, Sander J, Xu X. A Density-Based Algorithm for Discovering Clusters in Large Spatial Databases with Noise. In: Simoudis E, Han J, Fayyad UM, editors. Second International Conference on Knowledge Discovery and Data Mining. AAAI Press; 1996. p. 226–231. Available from: https://citeseerx.ist.psu.edu/viewdoc/download;jsessionid=9E6FE78EE86D98E605DEDBAF0582CF09?doi=10.1.1.121.9220&rep=rep1&type=pdf.
