# Supplementary material for: Improved prediction of hiking speeds using a data driven approach
Source: PLoS One. 2023 Dec 18;18(12):e0295848. doi: 10.1371/journal.pone.0295848 (PMC10727444; doi:10.1371/journal.pone.0295848)
Supplement: S3 File — (PDF) [file pone.0295848.s003.pdf]

S3 Supporting Information. Exploratory data modelling study

Data from Scotland was used for exploratory testing of different modelling approaches. When doing this, an earlier iteration of the break finding and data filtering process was used. Data were processed as described in S2 Supporting Information, with the following exceptions:

- GPS track segments had to be fully contained in the reduced 'Scotland' OS grid tiles described in S2 Supporting Information.
- Track segments where the median speed was greater than 10 km/h were not automatically removed prior to break identification.
- Individual points representing 10 minutes of travel were tagged as breaks (3 minutes was used in later versions).
- High speed (>10 km/h) points occurring immediately following a long (>3 minute) point were not automatically tagged as breaks.
- When merging data into 50 m sections, sections under 50 m in length immediately preceding a break or the end of the segment were also tagged as breaks, rather than combined with the previous section.
- After merging the data into 50 m sections, any section with a speed above 10 km/h which was at the start or end of a segment, or next to a break point were only considered to be part of the break in the OSM data, not the Hikr data.
- A minimum distance of 250 m of travel was included between breaks. Sections with a distance below this were tagged as a break.
- When looking for 'key points' to filter out non-walking track sections, the duration required to be tagged as a 'key point' was 10 minutes (3 minutes was used in later versions).
- Duplicate track segments were not identified or removed.
- The fastest and slowest 0.5% of the merged datapoints were not removed as outliers.

Once the data were filtered and processed, we were able to use them to test models for the walking speed. Two different approaches were explored in order to model the data: a generalised linear model (GLM) and a generalised additive model (GAM).

We know that predictions for walking speeds must be non-negative, and two different setups were explored to achieve this: a Gaussian distribution with log link function and a Gamma distribution with inverse link function. The GAM approach was also deployed with both thin plate spline or cubic regression basis functions. Investigations into different models showed that there was no improvement to model fit beyond cubic terms in a GLM, or 7 knots in each GAM smoothing term, so more complex models than this were not considered for selection.

Both model types were created in R version 3.6.1:

$$\texttt{glm}(v \sim a\phi + b\phi^2 + c\phi^3 + d\theta + e\theta^2 + f\theta^3, \texttt{distribution})$$
$$\texttt{gam}(v \sim s(\phi, k, \beta) + s(\theta, k, \beta), \texttt{distribution})$$

where

v = walking speed
ϕ = hill slope angle (degrees)
θ = walking slope angle (degrees)
k = knots used in spline (up to 7)
β = basis function

Initially, 10-fold cross-validation was used to compare the model parameters, looking at R-squared values, root-mean-squared error (RMSE) and mean absolute error. All models produced very similar results, with no change in the RMSE to 2 decimal places, although there was a general trend of marginal improvements as the model complexity increased. As no best model could be chosen based on the cross-validation, each was checked in more detail. Firstly, the hill slope component was isolated by investigating the speed when the walking slope was zero (i.e when traversing across a slope). Intuitively, and from experience, this should be a decreasing function; as the slope gets steeper it is harder to traverse, so the walking speed will decrease. Models which failed to predict this were removed under the assumption that the data were overfitted. Following this, the walking slope component was investigated, specifically looking at the walking speed when travelling directly up- or down-hill. By inspection of the data, existing functions, and intuition, this should be modelled as a roughly bell-shaped function with the peak at, or close to, 0 degrees. Any models which predicted an increase in speed as walking slope steepness increased (from a minimum magnitude of 10 degrees) were removed. Secondly, we know from existing work that there exists a critical gradient at a walking slope of around 15 – 21 degrees, at which it becomes more efficient to zig-zag up a steep hill rather than going directly uphill. Models which failed to predict the critical gradient occurring below 21 degrees when travelling uphill were also removed.

This resulted in 21 model configurations remaining, although it is clear from Fig 1 that the speed predictions are very similar in most circumstances. Fig 1A shows that all of the remaining models predict very similar speeds when traversing a slope of up to 40 degrees, after which there is more deviation in predictions. Similarly, when travelling in the slope direction (Fig 1B), all of the models are broadly similar on slopes up to approximately ± 15 degrees. More than 96% of the data is contained within this area, and the relative lack of data outside this region explains the divergent speed predictions. As all of the models provided both very similar R-squared values and very similar predictions over the vast majority of the dataset, we used the following points to make our final selection:

- It is easier to apply GLMs than GAMs to future work, as a simple formula to predict the walking speed can be produced for application elsewhere, without needing to recreate the model from the original data.
- In general, simpler models are easier to interpret, and we had no clear evidence that a more complex model would perform better.

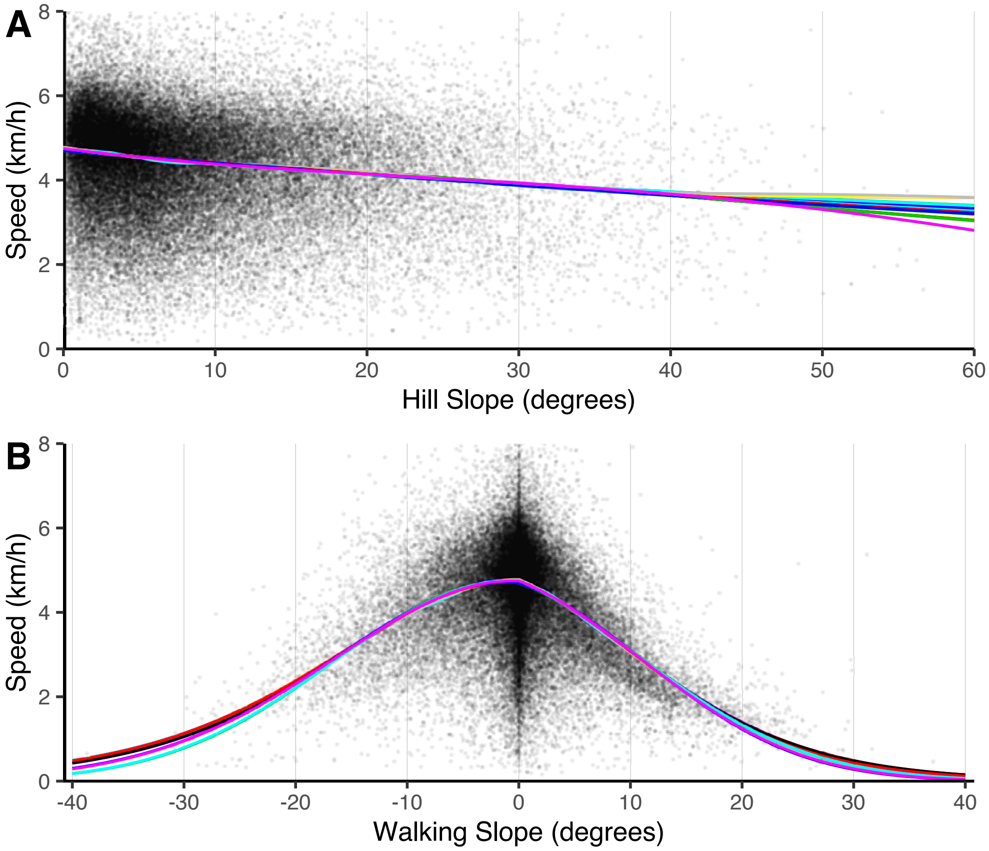

Fig 1. Walking speed predictions from 21 possible models (coloured individually) generated from the Scotland GPS dataset. (A) Walking speed predictions for traversing across hills of varying slope, overlaid on GPS data where walking slope is below 5 degrees. (B) Walking speed predictions for travelling directly up or down hills of varying slope, overlaid on GPS data where walking slope is within 5 degrees of hill slope.
