# Supplementary material for: Improved prediction of hiking speeds using a data driven approach
Source: PLoS One. 2023 Dec 18;18(12):e0295848. doi: 10.1371/journal.pone.0295848 (PMC10727444; doi:10.1371/journal.pone.0295848)
Supplement: S4 File — (PDF) [file pone.0295848.s004.pdf]

S4 Supporting Information. Exploring the differences between Scotland and the rest of the UK

When applying the GLM formulation separately to datasets covering Scotland and the rest of the UK (ROUK) (Fig 1), we see faster predicted walking speeds in the ROUK model than in the Scotland model when traversing a slope, or when walking uphill. Before building a model on a combined dataset, we wanted to check whether the Scotland dataset was a reasonable subset of the ROUK data.

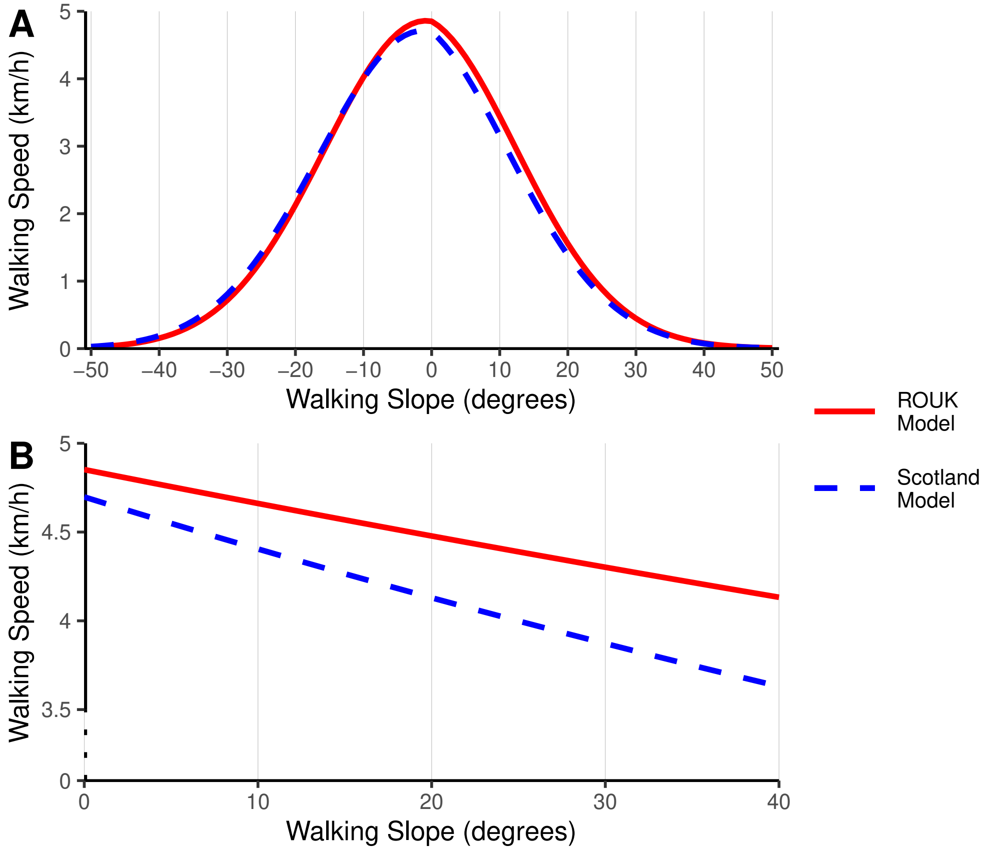

Fig 1. Comparison of walking speed models produced using data from Scotland and the rest of the UK. Walking speed predictions when: (A) travelling directly up or down hills of varying slope, (B) traversing across hills of varying slope.

To take into account the fact that the ROUK datset was much larger than the Scotland dataset (7636 tracks vs 648), we took 100 samples of 650 tracks from the ROUK dataset (to form sample sets of comparable size to the Scotland data) and modelled the walking speed for each one. The results are visualised in Fig 2.

When we look at traversing a hill, it is clear that the two datasets are distinct, as the model for Scotland is outside the range of results seen in the ROUK sample models. Further investigations showed that there were differences between the proportions of paved road, unpaved road and off-road data within each set. To take this into account, we modelled these individually, once again sampling the ROUK dataset 100 times to form sample sets of comparable size to the Scotland dataset (sample sizes of 600 for paved roads and 450 for unpaved and 200 tracks for off-road), and the resulting models can be seen in Fig 3.

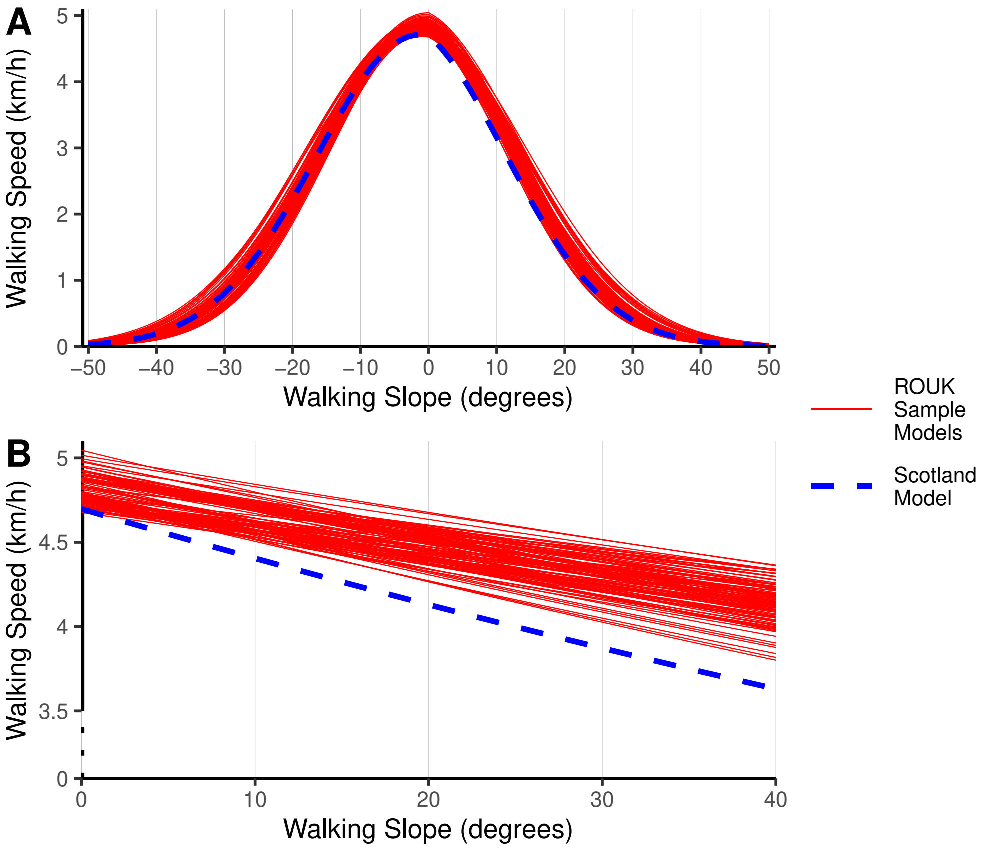

Fig 2. Comparison of walking speed models produced using data from Scotland against 100 sampled datasets from the rest of the UK. Walking speed predictions when: (A) travelling directly up or down hills of varying slope, (B) traversing across hills of varying slope.

We can clearly see now that our model for paved roads in the Scotland data is comfortably within the range of samples of the ROUK data. It is reasonable to suggest, therefore, that there is no difference in walking on a paved road in Scotland compared to the rest of the UK. However, our unpaved road model and off-raod models for Scotland lie at the extreme edge, or outside of the range of sample models taken from the ROUK unpaved data.

Before finally determining that the two datasets were distinct, we wanted to see if we could find another variable which would account for the lower walking speeds seen in Scotland on both unpaved roads and when off-road. We explored whether this elevation could be responsible for this difference, as higher elevations are likely to have higher exposure, and be more affected by inclement weather, leading to slower walking speeds.

There was a much greater proportion of data at high elevation (>500 m) in the Scotland dataset than the ROUK dataset on both unpaved roads and when off-road, while a silimar difference was not seen on paved roads (where our models were equal) - Fig 4.

For this reason we included elevation as a model variable, both as a continuous variable or as a factor variable classifying all points as either high elevation or low elevation (where high elevation consisted of all data >500 m). However, in both cases we found this to not be a significant factor in the model. Based on the sample data taken, we suggest that the model formulated using the Scotland data is an extreme

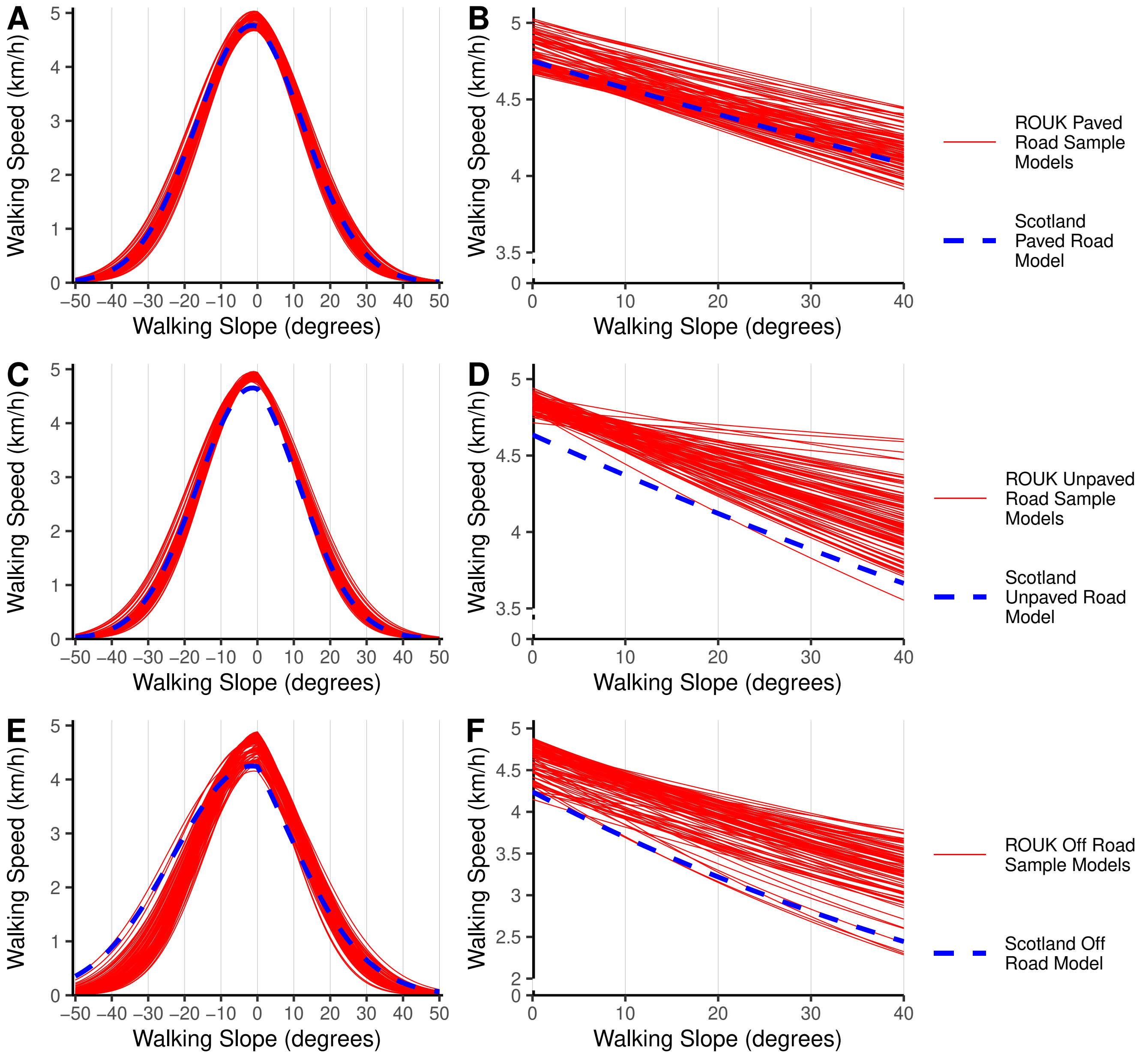

Fig 3. Comparison of walking speed models produced using data from Scotland against 100 sampled datasets from the rest of the UK. Walking speed predictions when: (A) travelling on paved roads directly up or down hills of varying slope, (B) traversing on paved roads across hills of varying slope, (C) travelling on unpaved roads directly up or down hills of varying slope, (D) traversing on unpaved roads across hills of varying slope, (E) for travelling off-road directly up or down hills of varying slope, (F) traversing off-road across hills of varying slope.

sample of the ROUK data, where a greater-than-average portion of the data has been sampled from high elevation regions. However, the high elevations themselves are not the cause of the difference between the model coefficients.

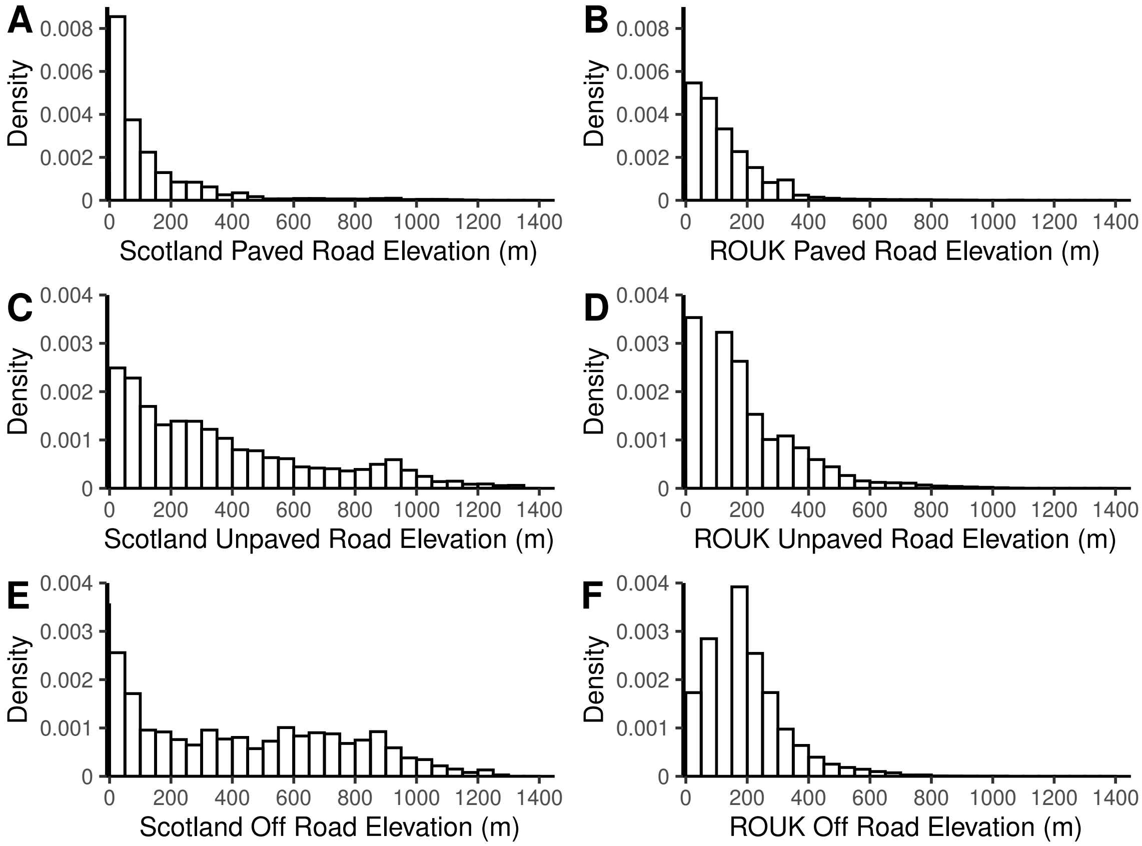

Fig 4. Comparing elevations of track sections between Scotland and the rest of the UK. (A) Scotland paved roads. (B) ROUK paved roads. (C) Scotland unpaved roads. (D) ROUK unpaved roads. (E) Scotland off-road. (F) ROUK off-road.
