# Supplementary material for: Improved prediction of hiking speeds using a data driven approach
Source: PLoS One. 2023 Dec 18;18(12):e0295848. doi: 10.1371/journal.pone.0295848 (PMC10727444; doi:10.1371/journal.pone.0295848)
Supplement: S5 File — (PDF) [file pone.0295848.s005.pdf]

S5 Supporting Information. Exploring the impact of terrain obstruction

To understand how terrain obstruction might impact the walking speed (and thus how it should be incorporated into a model), we conducted an initial exploration into the data. Before doing this, however, we wanted to check that there was not a systematic difference between the walking speeds in regions where we had lidar data, and regions where we did not. If the two regions were not found to be different, then any findings about the effects of terrain obstruction in regions where we had lidar data could also be applied to areas where we didn't have the data.

When modelling the data for the separate datasets ('obstruction available' vs 'no obstruction available'), we see that the models are very similar when ascending or descending a slope (Fig 1A). This was not the case when traversing the slope however, as the 'no obstruction available' model predicts that hill slope has a greater impact on reducing walking speed than the 'obstruction available' data model (Fig 1B).

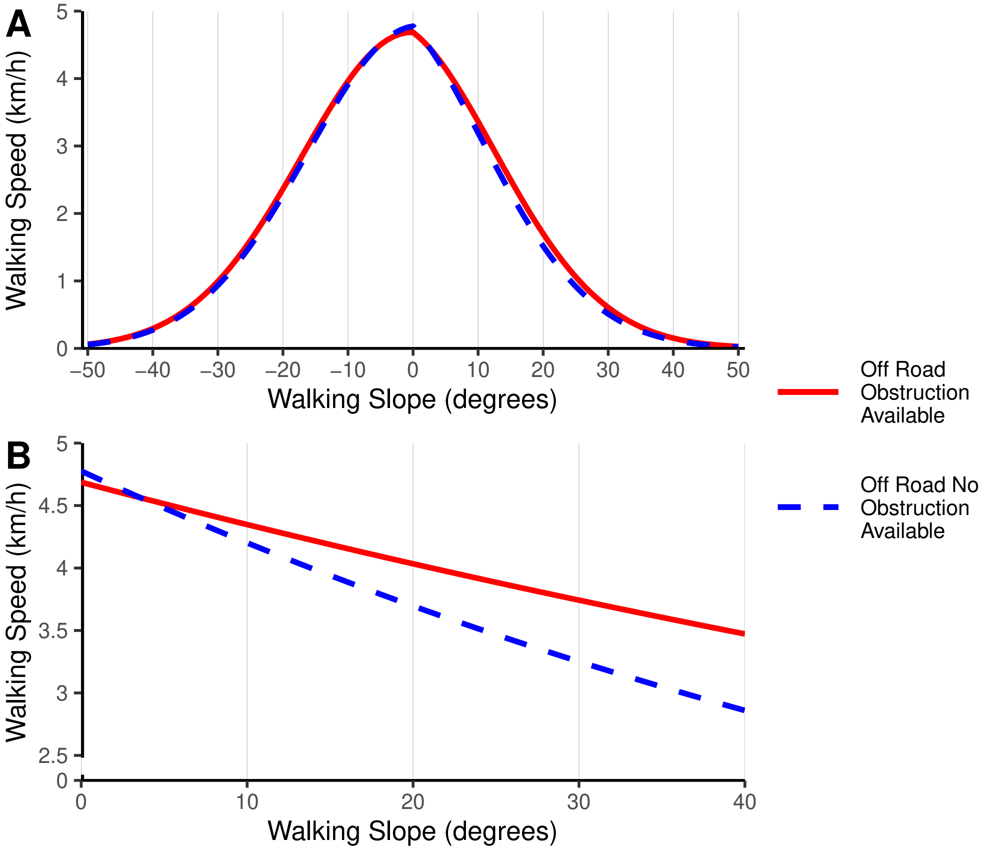

Fig 1. Comparison of off-road walking speed models where obstruction data is, or is not, available. Walking speed predictions when: (A) travelling directly up or down hills of varying slope, (B) traversing across hills of varying slope.

We sampled our larger ('obstruction available') dataset, so that we had a similar number of tracks as in our smaller dataset, and compared models made from more equal volumes of data. When doing this (Fig 2), we found that the 'no obstruction available' model is within the range of sample models for traversing the slope, albeit at an extreme end. This is likely due to the low volume of data which we had at high

hill-slopes. (Only 50 km of data had a hill slope greater than 15 degrees with no lidar data available, and only 130 km with lidar data available). Going forward, we assumed that the regions where we had lidar data were representative of all off-road regions, and so any findings could be applied to both areas.

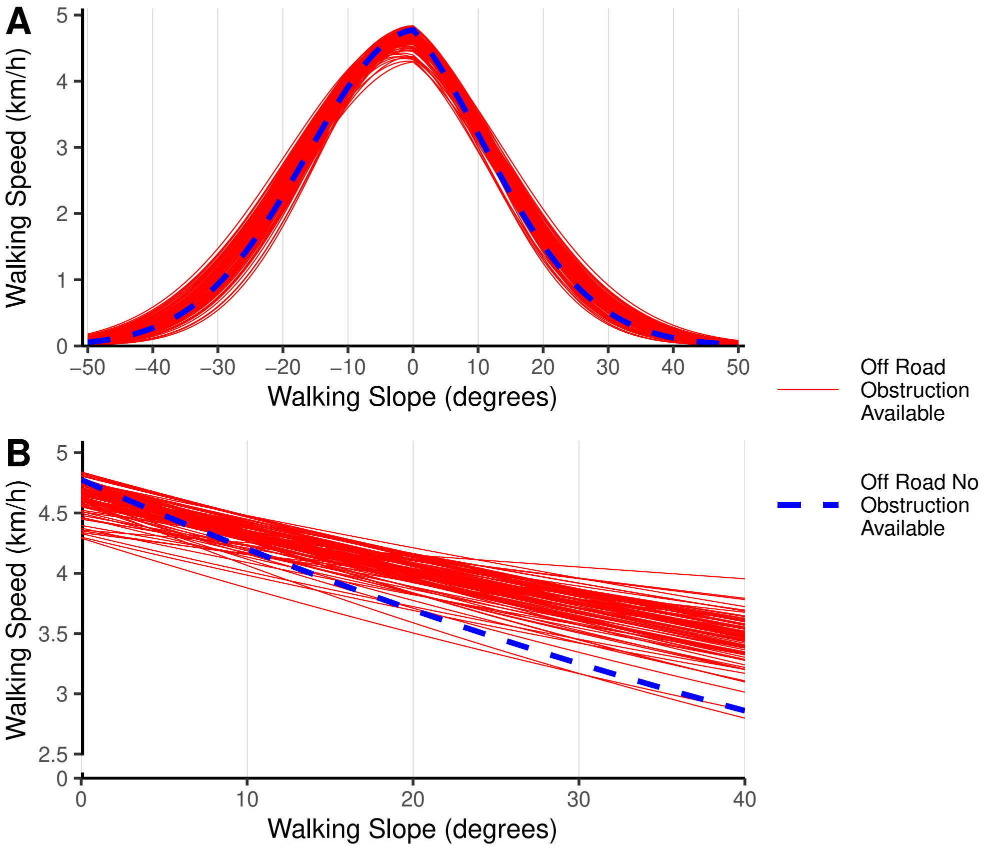

Fig 2. Comparison of off-road walking speed models produced using a dataset where obstruction data isn't available against 100 sampled datasets where obstruction data is available. Walking speed predictions when: (A) travelling directly up or down hills of varying slope, (B) traversing across hills of varying slope.

To explore the effects of terrain obstruction, we first looked at the range of speeds across the different obstruction values. The data was split into 25 quantiles, and the average walking speed for each was calculated. The results are shown in Fig 3A. This shows us two things; firstly the vast majority of our data had very little, or no obstruction (as most of the quantile points occur below 0.5 m of obstruction). Secondly we can see that there is a very steep drop off in walking speed initially, and it then remains relatively constant across obstruction levels. Our initial assumption was that walking would be relatively easy with no, or very little obstruction, and then much slower at obstruction values of approximately 0.5 m - 4 m when it would involve walking through thick vegetation, before getting slightly faster again at higher obstruction values (as you would be walking through a forest and could walk between the trees below the canopy). The data shows this not to be the case, although this may be a result of our data only showing us regions where walking was possible. Due to the crowdsourced nature of our GPS tracks, we had no data showing us the walking speed when in 2 m of thick vegetation, as it is very unlikely that people would have chosen to walk there.

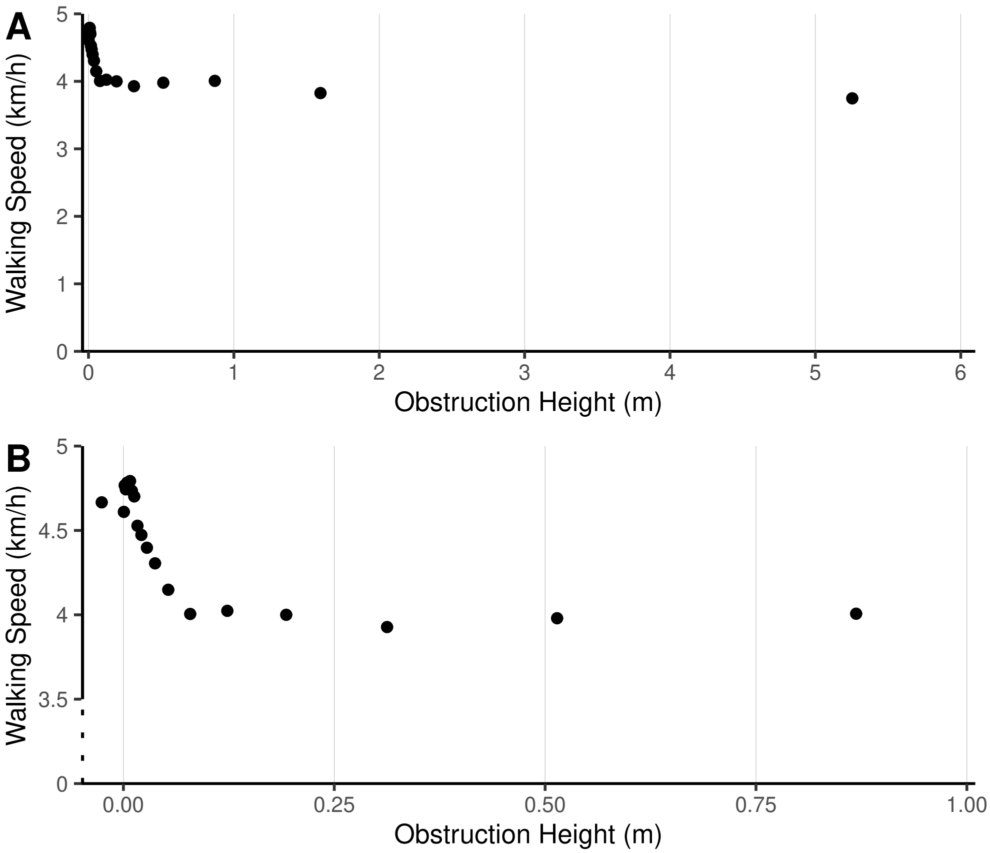

Fig 3. Binned average walking speeds across different levels of obstruction. (A) Full range. (B) Zoomed range. Each bin contains 1/25th of the datapoints.

Fig 3B shows a close-up of the steep speed drop off, and we can see that the average speed dropped from approximately 4.8 km/h when there was no obstruction down to about 4 km/h once there was more than 10 cm of obstruction. We used this information to classify all points into heavy obstruction (>10 cm) or light obstruction (<= 10 cm). Although the figure suggests a gradual decrease in walking speed between 0 and 10 cm of obstruction, we chose not to model this. Vegetation length is highly variable throughout the year, and it is more practical to classify regions as light or heavy obstruction when discussing walking speeds.
