# Supplementary material for: Improved prediction of hiking speeds using a data driven approach
Source: PLoS One. 2023 Dec 18;18(12):e0295848. doi: 10.1371/journal.pone.0295848 (PMC10727444; doi:10.1371/journal.pone.0295848)
Supplement: S6 File — (PDF) [file pone.0295848.s006.pdf]

S6 Supporting Information. Comparison of walking speed changes while crossing a simulated off-road terrain region

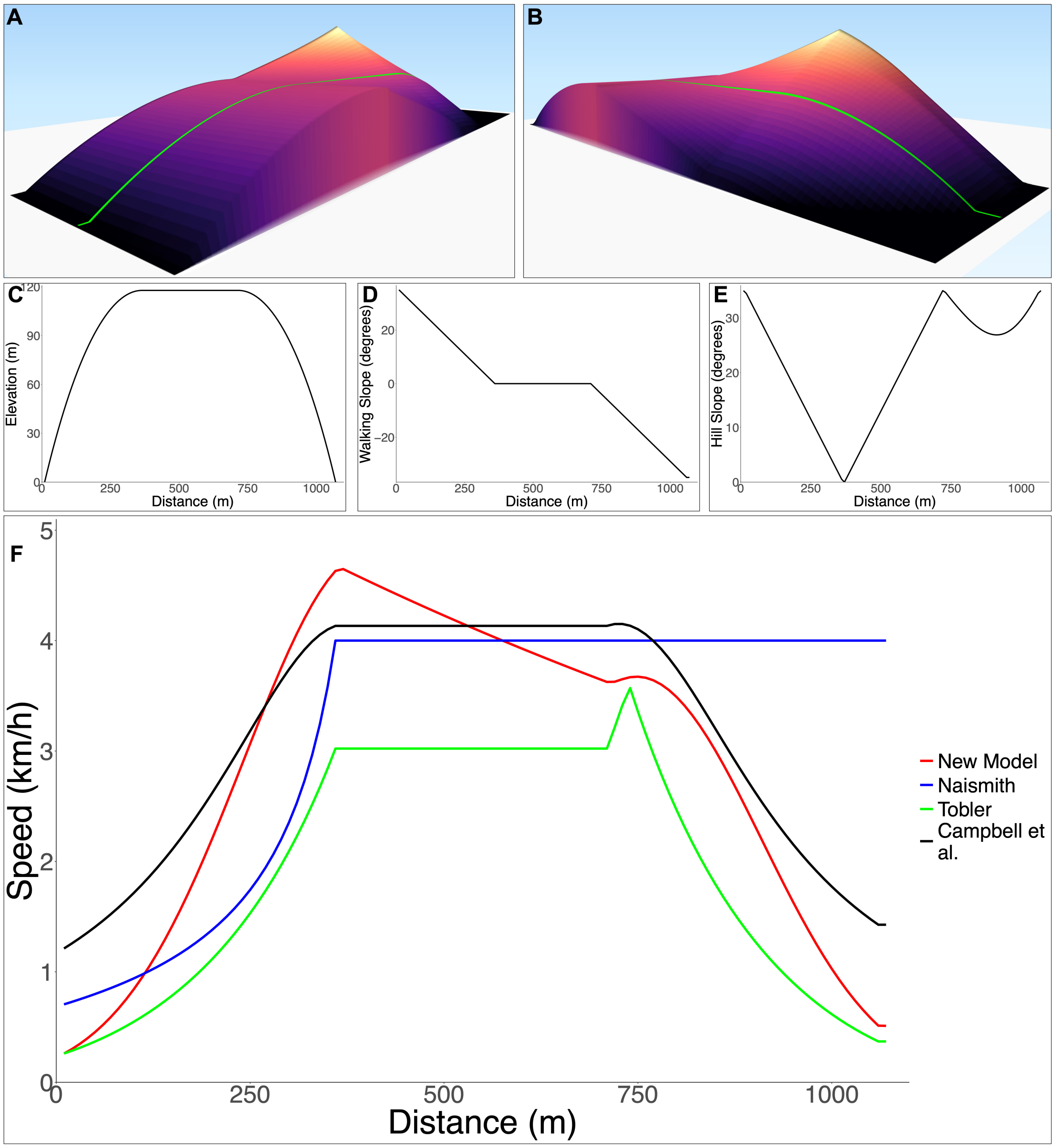

Fig 1. Comparison of walking speed changes while crossing a simulated off-road terrain region. (A), (B) The simulated route (green) across the terrain. Terrain is coloured by elevation value from low (dark) to high (light). (C) The elevation profile of the route, (D) The walking slope profile of the route, (E) The hill slope profile of the route. (F) Walking speed predictions for different models as the route is traversed. For Naismith's and Tobler's functions, the off-road variants of the models have been used. For the new model, the off-road unknown obstruction coefficients have been used.
