# Supplementary material for: Improved prediction of hiking speeds using a data driven approach
Source: PLoS One. 2023 Dec 18;18(12):e0295848. doi: 10.1371/journal.pone.0295848 (PMC10727444; doi:10.1371/journal.pone.0295848)
Supplement: Attachment — Submitted filename: ResubmissionResponseToReviewers.docx [file pone.0295848.s008.docx]

We would like to thank the reviewers for their additional comments and appreciate the improvements that these have made to the paper.

Statistical Significance of Model Improvement: One critical concern, previously highlighted in the last round of review comments, pertains to the practical significance of the model's performance. Given that the model incorporates multiple variables and employs Generalized Linear Models (GLM) for regression, the reported improvement achieved by the model appears to be modest. This issue has also been noted by other reviewers, particularly concerning the low R-squared value of 0.09. The authors should not overlook this concern and should provide a more thorough discussion of the model's practical utility and limitations.

The low R-squared indicates that there remains variability in the data that is not captured in the model. We have acknowledged this from the beginning by indicating that we know of many variables which will impact the speed (such as group ability etc), for which there are no data available to include in the model. This has been made more explicit at the end of the introduction [lines 99-101]. Further acknowledgement of this has also been added to the results [lines 288-292], and the following has been added to the discussion [Lines 356-361]: “The existing methods to calculate walking speeds require tuning for use in real-world scenarios, as there are many factors which can affect an individual's walking speed beyond the slope and obstruction level (such as the weather, fitness level or age). The model presented here requires the same tuning as these existing methods but provides more a more accurate population average walking speed across a wide range of terrain and slope conditions.”

Detailed Presentation of Data-Driven Approach: As the paper focuses on a data-driven approach to modeling walking speed, it would greatly enhance the understanding of the methodology if the authors provided more quantitative characteristics of the original data. For instance, Figure 3 illustrates walking speed predictions under various terrain conditions. It is essential to include statistical characteristics of the actual speed data (e.g., average, standard deviation, or the 95% confidence interval) when individuals are traversing different types of terrain, derived from real datasets. Additionally, incorporating statistical characteristics of the ground truth data in Figure 4 would strengthen the paper's credibility and the evaluation of the model's performance.

A new table (Table 1) and figure (Figure 4) have been added which illustrate the breakdown of the tracks by slope angle, and the confidence intervals for the mean walking speed. These demonstrate how our model closely fits the average walking speed, particularly in areas where the majority of walking occurs (where we have the most data), and thus where accuracy is most important. We have also added an explanation for the deviation from the confidence interval at high slopes [lines 260-272]

Transparency in Exclusion of Data Points: It would be beneficial for readers to know the number and percentage of data points or sections that were excluded due to the filtering process. This transparency will provide a clearer picture of the data selection and processing steps.

Responding to this point is tricky as “data points” were not truly defined or counted until after the initial filtering and merging. This means that there are no equivalent numbers for data points present in the unfiltered data that would support a direct comparison. However, we support the need for transparency and therefore all code used to filter the data is included for checking. The initial number of GPS tracks (~20,000) has been included in both Supplementary Information 2, and in the results. This reduced to ~7600 after processing (also mentioned in the results). The majority of the removed data was from non-walking tracks (mostly clear from the velocity), but a detailed breakdown of exclusions by filtering reasons was not recorded. The main details of filtering are in Supplementary Information 2 but the results section has been changed to make the need for significant filtering clearer. [Lines 217-220]

Additional Minor Comments:

Introduction References: In the introduction section, consider providing more references when discussing the classification of factors that can impact walking speed into two groups. This will enhance the depth of the literature review and provide a stronger foundation for your work.

Further references have been added, which discuss the factors which can affect walking speeds. [Line 15]

Model Performance Evaluation: Highlighting the fact that the model's performance evaluations are conducted at the section level should be done earlier in the paper to prevent any misunderstanding. Additionally, consider presenting statistical characteristics of the sections, such as their length and the distribution of slopes, to provide a comprehensive assessment.

A line has been added to the start of results, with the minimum and average point distance. [lines 222/223]. Table 1 has been added detailing the slope distributions of the data

Clarify Terminology: Distinguish between "hill slope" and "walking slope" throughout the paper to avoid confusion. For instance, in the caption for Figure 3, specify the meaning of "slope" in Figures 3A and 3B to enhance clarity for readers.

Figure captions have been adjusted as requested.
